# Influence of Ion Nitriding on Microstructure and Properties of Haynes 282 Nickel Superalloy Specimens Produced Using DMLS Technique

**DOI:** 10.3390/ma16145020

**Published:** 2023-07-15

**Authors:** Ryszard Sitek, Krzysztof Kulikowski, Krystian Paradowski, Kamil Gancarczyk, Monika Losertová, Akira Kobayashi, Joanna Moneta, Janusz Kamiński

**Affiliations:** 1 Faculty of Materials Science and Engineering, Warsaw University of Technology, Woloska 141, 02-507 Warsaw, Poland; 2 Department of Materials Science, Faculty of Mechanical Engineering and Aeronautics, Rzeszow University of Technology, Al. Powstancow Warszawy 12, 35-959 Rzeszow, Poland; 3 Department of Materials Engineering and Recycling, Faculty of Materials Science and Technology, VSB—Technical University of Ostrava, Ostrava, 17. listopadu 2172/15, Poruba, 708 00 Ostrava, Czech Republic; 4 Department of Physics, Faculty of Science, Chulalongkorn University, Bangkok 10330, Thailand; 5 Department of Aeronautics and Astronautics, Faculty of Engineering, The University of Tokyo, Bunkyo-ku, Tokyo 113-8656, Japan; 6 Institute of High Pressure Physics, Polish Academy of Sciences, Sokolowska 29/37, 01-142 Warsaw, Poland

**Keywords:** haynes 282 nickel superalloy, direct metal laser sintering, ion nitriding, corrosion resistance, tensile test, microstructure

## Abstract

The paper investigates the influence of the ion-nitriding process on the microstructure, corrosion resistance, and tensile strength at elevated temperatures of Haynes 282 nickel superalloy specimens produced by the Direct Metal Laser Sintering (DMLS) technique. The study was performed for two conditions, i.e., as-built by DMLS method and as-built by DMLS method + covered by a layer containing CrN + Cr_2_N phases. An analysis of the surface morphology revealed that the ion-nitriding process significantly affects the physical and chemical phenomena occurring on the specimen’s surface. The XRD measurement of the specimens showed that preparing them with the DMLS method as well as following a nitriding process produced residual tensile stresses. Based on the measurement of the nanohardness distribution through the layer approximatively of 7 μm in width and the superalloys substrate, the results of the nanohardness showed the maximum values of 27 GPa and 13.5 GPa for the nitrided layer and the substrate, respectively. The surface protection from the nitrided layer proved a positive effect on the corrosion resistance of the DMLS specimens in the solution of 0.1 M Na_2_SO_4_ + 0.1 M NaCl at room temperature. The results of the tensile tests at 750 °C showed that the ion-nitriding process did not significantly affect the elevated-temperature tensile strength of the superalloy specimens produced with the DMLS technique.

## 1. Introduction

Nickel-based superalloys are widely used in various spheres of application, e.g., energy, aerospace, and oil or gas processing industries [1,2,3,4,5]. Their versatility results from both good mechanical properties and corrosion resistance (high temperature, electrochemical) in different aggressive environments [6,7,8].

One of the newest nickel superalloys developed for high-temperature application in aggressive environments is Haynes 282 nickel superalloy (hereinafter 282 alloy) [9,10]. This multiphase superalloy based on Ni-Cr-Co-Mo-Al-Ti is mainly formed of γ matrix (fcc structure) strengthened by γ’-Ni_3_Al phase (ordered L1_2_ structure) and MC and M_23_C_6_ carbides [11]. The high strength properties and good weldability of 282 alloy [12] result in carefully defined aluminum and titanium contents, which affect the volume fraction of the γ’ phase. Generally, the high content of γ’ particles in wrought superalloys significantly limits the formability in a subsequent treatment process [11], especially for the superalloys produced by means of additive manufacturing techniques.

Laser Beam Powder Bed Fusion manufacturing techniques (LB-PBF), for example, Selective Laser Melting (SLM) or Direct Metal Laser Sintering (DMLS), are increasingly used in the producing of metallic parts from various alloys, such as Ni-based superalloys, stainless steels or titanium materials [13,14,15,16,17,18,19]. These processes in which layer after layer of metal powder are selectively melted by the interaction of a high energy density laser beam offer many advantages over conventional manufacturing methods. Nevertheless, it should be emphasized that the processes produce non-equilibrium microstructures, residual stresses, and anisotropic properties that are caused by high-temperature gradients. Some negative effects that appear during the building of the net-shaped parts with complex geometries can be reduced mainly with process parameters, such as laser power, beam diameter, hatch distance, layer thickness, scanning velocity, and scanning strategy.

The negative effects are mainly caused by the crystallographic texture, the structure of melt pools, the privileged distribution of certain phases and carbides, and the presence of asymmetrical defects [19,20]. Boswell et al. in their study [20] showed that the anisotropic effect could be effectively reduced with appropriate dissolution and ageing heat treatment, leading to recrystallization and local texture reduction. Applying a high temperature to the building platform during the LB-PBF process has also been shown to be effective in reducing adverse anisotropy effects, such as grain-boundary cracking, associated with accumulating γ’ and carbide precipitates [21].

The service life of the turbine blades produced of superalloys can be shortened through oxidation or hot corrosion. The passive oxide layer which spontaneously forms on the surface significantly increases corrosion resistance compared to the substrate in the active state [22]. The study in [23] concluded that 282 alloy showed good static oxidation resistance at testing temperatures of 871, 927, and 982 °C; these results are comparable with other γ’-Ni_3_Al-strengthened superalloys such as 263 alloy and WASPALOY alloy. Nevertheless, erosive effects of hot gases or gas stream that can include impurities and particles may erode and destroy protective scales on the surface. Further, an oxide cathode layer makes the passive oxide layer susceptible to local corrosion in the presence of aggressive ions, such as chloride (Cl^−^) or sulphate (SO_4_^2−^) ions, that are often present in many industrial environments [24].

In the 1960s, the first protective layers on the superalloys were applied after extensive research to increase high oxidation resistance and high stability as well as to achieve a good combination of physical and mechanical features. These include the first diffusion aluminide coatings, overlay coatings, thermal barrier coatings (TBC), or relatively new coating produced with nitriding processes [8,25,26,27,28,29,30,31,32,33].

Ion-nitriding processes belong to modern thermo-chemical treatments enabling the production of protective diffusion layers with controlled phase composition and high-performance properties. Such surface layers contain chromium nitrides, i.e., CrN and/or Cr_2_N, characterized by high hardness and resistance to corrosion, abrasive wear, or oxidation. These diffusion layers or coatings have been successfully produced on some steels [34,35,36,37]. However, there is a lack of information on the ion nitriding of nickel superalloys, in particular, Ni-based superalloys having the characteristics of a strongly defective anisotropic structure. The similarity in the structure (fcc) of austenitic stainless steels and Ni-based superalloys can lead to similar results in the investigation of their susceptibility to localized corrosion and poor tribological performance.

The CrN coatings exhibit the higher wear resistance whereas the Cr_2_N films show the better hardness. Nitrided coatings have an inert intrinsic behavior either in acidic or in chloride solutions. However, their capability of protecting the substrate from any corrosion is limited due to defects and porosity [29]. In the case of nitrided layers produced on Ni-Cr alloy [30] exposed in chloride-free environments (H_2_SO_4_), the CrN + Cr_2_N layer is not susceptible to pitting corrosion. In addition, it has been shown that the best corrosion resistance is achieved when (CrN + Cr_2_N) phases are combined rather than when a homogeneous Cr_2_N or π (Cr_12.8_Ni_7.2_N_4.0_) phase is used alone. It is assumed that the differentiated corrosion resistance of chromium nitrides is related to the different crystallographic structures of the CrN (fcc A1) and Cr_2_N (hcp A3) phases and the packing density of atoms in the unit cell.

In nitride layers formed on alloys rich in Cr atoms, it is also possible to have negligible amounts of other phases (α-Cr, Cr_2_O_3_) that can further reduce the corrosion resistance of the substrate.

When the nitriding process of Ni-based alloys is performed with gaseous or plasma transport media at temperatures above 450 °C for a long duration, the formation of CrN precipitates in a Cr-depleted fcc–Ni matrix has been demonstrated [32,33]. Nevertheless, a fcc phase supersaturated with nitrogen in austenitic stainless steels (i.e., expanded austenite γ_N_) was shown using a low-energy ion implantation at lower temperature (below 400 °C) [38,39]. Similarly, this supersaturated phase in Ni-based superalloys could combine high hardness and good corrosion resistance. From this point of view, the study of nitriding is of great interest for enhancing the current knowledge of the influence of the process on transformations and final properties of Ni-based superalloys.

In this paper, the properties of the substrate prepared from 282 alloy powder with the DMLS method was studied in the context of the microstructure in the as-built and as-built + nitrided conditions. The influence of microstructure on the corrosion, hardness, and tensile behavior at an elevated temperature was studied and compared to wrought 282 alloy. The present investigation on 282 alloy is a follow-up to our earlier work [40].

## 2. Materials and Methods

### 2.1. Specimens’ Preparation Using the DMLS Technique

The specimens investigated were prepared from the Amperprint^®^0233 Haynes^®^ 282^®^ powder purchased from Höganäs firm. The typical chemical composition of the alloy is shown in Table 1 [41]. The nominal diameter of the spherical particles ranges between 15 and 45 µm. The particle size and sphericity of the alloy 282 powder were investigated in our recent work [40]

The test specimens with dimensions of 12.2 × 12.2 × 40 mm^3^ were built using an EOS M100 printer equipped with a 200 W ytterbium fiber laser and operating in Direct Metal Laser Sintering (DMLS) technology. The building process of the specimens was performed with the following parameters: laser power P—90 W, scanning velocity V—800 mm/s, layer thickness—20 μm, hatch spacing H—0.05 mm, and under argon atmosphere of 99.999% purity. Each subsequent layer was scanned in a direction rotated by 67° relative to the previous layer.

In order to compare selected properties, the same testing specimens were prepared from wrought rods of Haynes^®^ 282^®^ alloy purchased from Haynes Int., Inc., the composition of which is listed in Table 2 [42].

### 2.2. Ion-Nitriding Process

**Figure 1:**
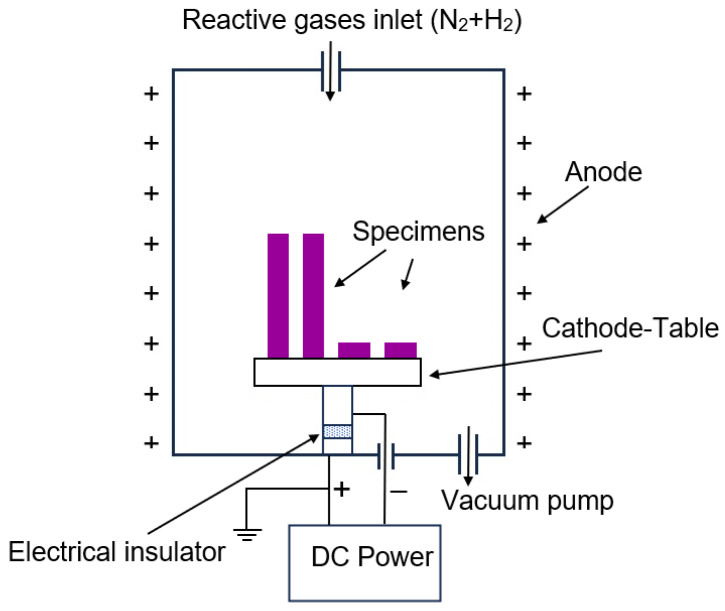
Schematic of a universal device for the ion-nitriding process.

Before the nitriding process, the surfaces of the specimens built with DMLS in the x-y plane were ground on abrasive SiC papers of the Grit 800, then were washed with ethyl alcohol in an ultrasonic scrubber, dried and placed in a universal ion nitriding furnace, the schematic of which is shown in Figure 1. Plasma/Ion Diffusion Treatment (PDT) process allows treating objects in a cathodic potential when the furnace wall works in an anodic potential. The ion-nitriding process was performed on the specimen surface parallel with the building direction in a mixture of 25% N_2_ + 75% H_2_ gases for 12 h at 570 °C and 3.5 hPa.

### 2.3. Microstructure

The microstructures of the specimens as-built (hereinafter 282–AB), as-built and nitrided (282–AB+N), and wrought 282 alloy (282–W) were investigated by means of light, scanning electron (SEM) and transmission electron (TEM) microscopies. All specimens were studied in the cross-section perpendicular on the building or wrought direction, respectively. The specimens were ground, polished, and etched using the reagent composed of 100 mL 38% HCl + 100 mL C_2_H_5_OH + 5 g CuCl_2_. The observation with TEM was performed only for 282–AB and 282–AB+N specimens using a FEI Tecnai G2 F20 S-TWIN operated at 200 kV. Cross-sectional TEM thin foils were prepared with mechanical polishing and subsequent Ar-ion milling until electron transparency using the PIPS system from Gatan, Inc. The surface morphology of the 282 alloy specimens after corrosion tests were also studied by means of light microscopy.

### 2.4. Nanohardness of the Nitrided Specimen

Nanoindentation tests were performed on polished 282–AB+N specimens using a Berkovich indenter on NanoTest Vantage Alpha by Micro Materials, Ltd. (Wrexham, Wales) The determined load-displacement curves as a function of the indenter displacement were analyzed according to the Oliver–Pharr method to evaluate nanohardness. Forty-one indentations were carried out across the nitrided layer and substrate in zig-zag displacement with 0.3 μm distance, 1 mN load, 10 s loading time, 10 s unloading time, and 5 s dwell period.

### 2.5. Corrosion Resistance Tests

Corrosion resistance tests of the 282–AB, 282–AB+N and 282–W specimens were performed in a non-deaerated solution of 0.1 M Na_2_SO_4_ + 0.1 M NaCl at room temperature by means of the AutoLab PGSTAT100 potentiostat. A conventional three-electrode cell inside a Faraday cage was used where the tested specimen served as the working electrode (WE), platinum as the reference electrode (RE), and saturated calomel electrode (SCE) as reference electrode. The surface of the WE exposed to the solution was 28.3 mm^2^.

Before electrochemical testing using impedance (EIS) and potentiodynamic (LSV) methods, the specimens were exposed to the solution mentioned above under electroless conditions for the 5000 s needed to stabilize the Open Circuit Potential (OCP). EIS tests were carried out in the frequency range of 10^5^ Hz–10^−3^ Hz, with sinusoidal signal amplitude of 10 mV in potentiostatic mode at OCP that was stated for 282–W = −233 mV, 282-AB = −211 mV, and 282–AB+N = +2 mV. Impedance spectra were analyzed using Baukamp’s EQUIVCRT program (4.9.007).

Potentiodynamic tests were carried out in an identical three-electrode system up to a potential of 1500 mV. To predict a possible hysteresis loop, a return polarization (inverted curve) was performed after anodic polarization. Regardless of the polarization direction, the potential change was 0.2 mV/s. Surface topography studies were carried out using the ACCURION optical profilometer HALCYONICS_i4 Sensofar Metrology (Barcelona, Spain) using the SensoVIEW program (1.8.0).

### 2.6. Analysis of the Phase Composition and Residual Stresses

The phase composition analysis was carried out using Rigaku’s (Auburn Hills, MI, USA) Miniflex II X-ray diffractometer (JPN). Filtered X-rays of the CuKα1 λ = 0.154 nm tube in the Bragg–Brentano diffraction geometry were used. XRD diffraction patterns in the 20°–100° 2θ range, with a 2θ-step of 0.02° and 3 s collection times. The phase composition was determined using the diffraction database PDF (Powder Diffraction File), developed by ICDD (The International Centre for Diffraction Data). The identification of phase components consisted of adjusting the profile of the obtained diffraction pattern, i.e., the calculated distances between hkl planes (d_hkl_) for individual reflections and their intensity to the data in the PDF database.

The residual stresses relating with the nitriding process were determined for the marked points on the surface of the 282–AB and 282–AB+N specimens. Proto and XRD Combo X-ray diffractometer (Taylor, MI, USA) and Proto Manufacturing (CAN) XRD Win 2.0 computer software were used. The measurement was performed using a lamp with a manganese anode MnKα1 λ = 0.210 nm, a collimator diameter of 2 mm, an anode current of 4 mA, and an anode voltage of 20 kV. To calculate the residual stress values at a given measuring point, the sin^2^Ψ method [43] was employed. This standard method involving the use of symmetrical Bragg–Brentano diffraction uses a goniometer of the Ψ type, which makes it possible to obtain the proper inclinations of the diffraction vector with angles Ψ and in a plane perpendicular to the diffraction plane [44]. Residual stresses were determined for constant values of the angle Ψ in the range from 25 to −25°. Elastic deformations in the tested specimens were applied for the diffraction line from the family of {311} planes at an angle of 2θ = 155.2°. For the residual stress measurements, Poisson’s coefficient value of ν = 0.27 and Young’s modulus value of E = 280 GPa were assumed [45].

### 2.7. Tensile Testing at 750 °C

Tensile behavior was tested at 750 °C by means of an electromechanical testing Microtest machine equipped with a force sensor in a measuring range of 50 kN and controlled by MICROTEST SCM3000 software. Testing at elevated temperature was assured by means of a cylindrical three-zone vertical furnace HT-ST 1000 operating in the range from 100 to 1000 °C with an accuracy of 0.1 °C controlled by the Eurotherm 2704 software. For each type of the investigated materials, i.e., 282–AB, 282–AB+N, and 282–W, two specimens were subjected to a tensile loading with the strain rate of 3.3 × 10^−4^ s^−1^. Due to the measuring base of the extensometer, the real measured length of the specimens was 25 mm. Based on the results obtained, the average values of mechanical properties were determined.

**Figure 2:**
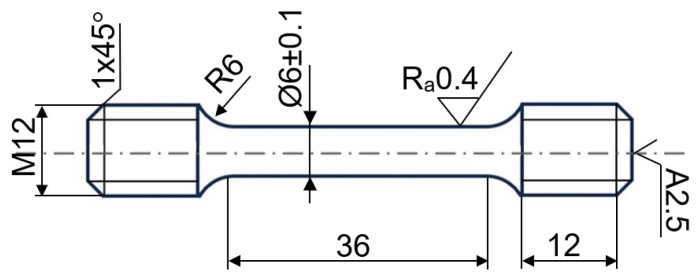
Schema of the tensile test specimen (in mm).

The geometry of axially symmetrical specimens with a circular cross-section of 6 mm diameter and a standard gauge length of 36 mm is presented in Figure 2.

## 3. Research Results and Discussion

### 3.1. Microstructure

**Figure 3:**
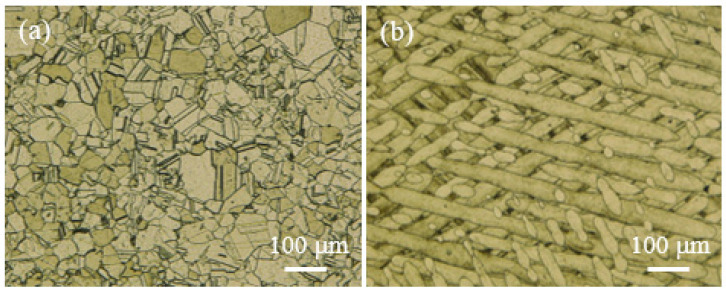
Microstructure of 282 alloy specimens: (**a**) 282–W, (**b**) 282–AB built in XY plane.

Figure 3 shows the microstructure of 282–AB and 282–W specimens. The microstructure of 282–W (Figure 3a) is formed of austenite grains and twins. For the microstructure of the 282–AB specimen, Figure 3b is typical in that layers/welds are arranged alternately corresponding to the laser-scanning strategy.

**Figure 4:**
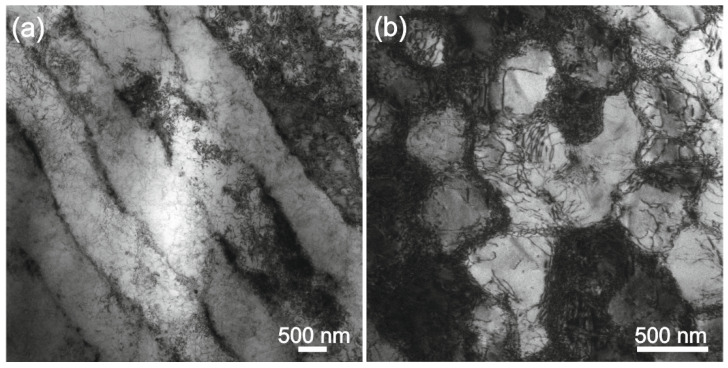
TEM images of 282–AB specimen with different areas in the cross-section perpendicular on the building direction: (**a**) elongated shape cells and (**b**) hexagonal shape cells.

Figure 4 shows TEM bright field images for the cross-sections perpendicular on the building direction of the 282–AB specimen. The images come from two different regions located at a distance of about 400 μm from the surface. The austenite grains, which are about a few micrometers in size, have a high density of dislocations. Dislocations form a regular cellular structure with cells of about 500–1000 nm. As the diffraction contrast shows, the cells are slightly misoriented, forming a sub-grain structure inside the austenite grains. TEM investigation revealed different sections through the cellular structure leading to the conclusion that the cells are elongated in one direction (Figure 4a) and adopt a hexagonal shape in an edge-on view (Figure 4b). As it was observed for additively manufactured austenitic steel [46], this kind of dislocation structure and the direction of the cell elongation can be attributed to the scanning direction of the laser beam. Since the scanning strategy consisted of changing the direction by 67° during the building, different cell orientations and shapes are remarked in the 282–AB specimen.

**Figure 5:**
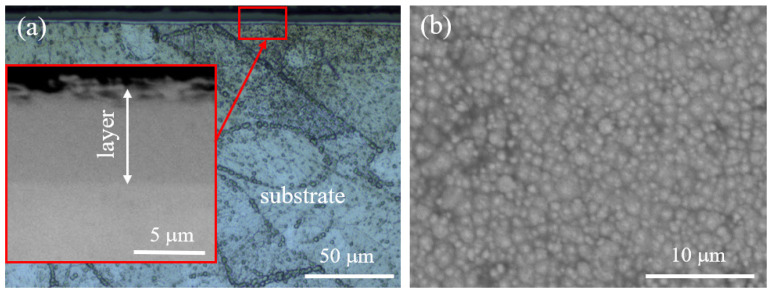
Nitrided layer on the 282–AB+N specimen: (**a**) the substrate/layer interface with the detailed surface film on the layer; (**b**) SEM surface layer morphology with very fine particles.

Figure 5 shows an optical micrograph of the nitrided layer on the 282–AB+N specimen and an SEM surface morphology of the layer. From the microscopic observations, it can be estimated that the thickness of the nitrided layer reaches approximatively 7 µm (Figure 5a). This layer seems to be uniform and continuous over the entire substrate/layer interface observed. No visible voids at the interface were detected. Very fine particles are observed on the surface film of the layer (Figure 5b).

### 3.2. Phase Analysis and Residual Stress

**Figure 6:**
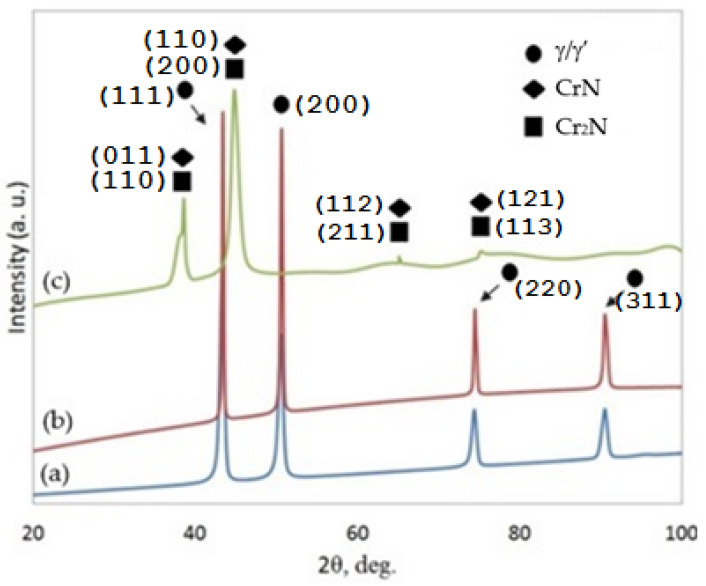
Diffraction patterns for the specimens: (a) 282–W, (b) 282–AB, and (c) 282–AB+N.

Analysis of the phase composition of the 282–AB and 282–W specimens showed very similar lines on diffraction patterns and the occurrence of phases: γ-Ni (PDF Card 01-077-9326) and/or phase γ’ (PDF Card 04-004-2742) (Figure 6). Due to the similar values of the lattice constants of the γ and γ’ phases, it is not possible to clearly distinguish from which phase the obtained reflections originate. The 282–W specimen that was prepared from purchased forged roads (i.e., after thermo-mechanical treatment) contained both phases [42], while the reflections obtained for the 282–AB specimen correspond to γ phase because the rapid cooling of the pools during SLM building and the lack of a post-processing heat treatment probably led to solid-solution appearance only. The ion-nitriding process changed the phase composition on the surface of the 282–AB+N specimens. Two nitride phases were found: CrN (PDF Card 04-007-0676) and Cr_2_N (PDF Card 00-035-0803). The CrN phase crystallizes in the orthorhombic structure (Pnmm), and the lattice parameters of the unit cell are a_0_ = 0.287, b_0_ = 0.297, and c_0_ = 0.4132 nm. In the others, the Cr_2_N phase crystallizes in the trigonal structure (P31m), with the unit cell lattice parameters equal to a_0_ = 0.481 nm and c_0_ = 0.448 nm.

Residual stress values were measured in 282–AB and 282–AB+N to determine the effect of the nitriding process on the as-built structure. In both cases, a tensile stress state was found. The highest tensile stress value of +152 ± 24 MPa was found for 282–AB, while the 282–AB+N specimen showed a lower value of +64 ± 45 MPa.

It can be assumed that the magnitude and nature of the stresses examined in the 282-AB specimen are mainly due to the physical and chemical properties of the alloy, the large temperature gradient during melting and cooling, and the adopted laser beam scanning strategy. As it was stated in [47], microstructures of the counterparts produced with the SLM technique from superalloys generally display an anisotropy and residual stresses. The research work in [48] also indicated that the values of residual stresses on the surface and in the volume of the specimen differ and depend on the adopted processing method. Therefore, to remove unfavorable residual stresses, materials produced using SLM or DMLS manufacturing techniques need to be subjected to post-process heat treatment [49,50,51]. In the case of surface tensile stresses, some mechanical surface treatments, e.g., shot peening, can be reduced [52,53]. Based on diffraction patterns in Figure 6, the ion-nitriding process applied on 282 alloy built with DMLS led to a partial stress relieving (by 88 MPa) on the surface of the 282–AB+N specimen that can positively affect the mechanical properties of the alloy.

**Figure 7:**
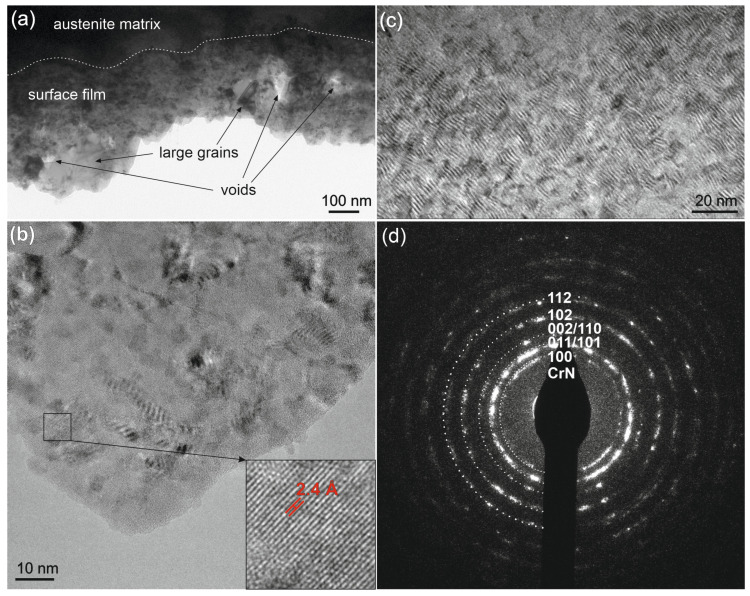
HRTEM images of the 282–AB+N: of (**a**) the surface film with CrN nanocrystals and underlying nitrided austenite matrix (the dotted line indicates the location of the interface); (**b**) detail of (**a**) with nanocrystals in the nitride film; (**c**) Moiré pattern of nanocrystals in the austenite matrix; (**d**) electron diffraction pattern of the nanocrystalline surface film in detail (**b**).

A TEM study of the 282–AB+N (Figure 7) revealed a thin surface film in the nitrided layer with an irregular thickness of about 200–400 nm. This film is formed of an amorphous matrix with very small grains of several nanometers and larger grains of tens to hundreds of nanometers (Figure 7a,b). Several voids were observed. In the underlying area of the surface film, austenite grains of about a few micrometers in size with a visible Moiré pattern were evidenced in the nitrided crystalline matrix (Figure 7c). The nanocrystalline surface film adheres well and passes smoothly to the underlying crystalline matrix. The presence of the nanograins precipitated in this area can be assigned to the CrN phase precipitated in the austenite matrix [54]. Electron diffraction analysis of the thin surface film (Figure 7d) indicates the formation of a CrN phase, but the presence of a Cr_2_N phase cannot be excluded due to similar interplanar distances. Unlike the alloy substrate with the cellular dislocation structure (Figure 4), the nitrided layer extending to a depth of about 7 μm does not exhibit a high density of dislocations.

### 3.3. Nanohardness

**Figure 8:**
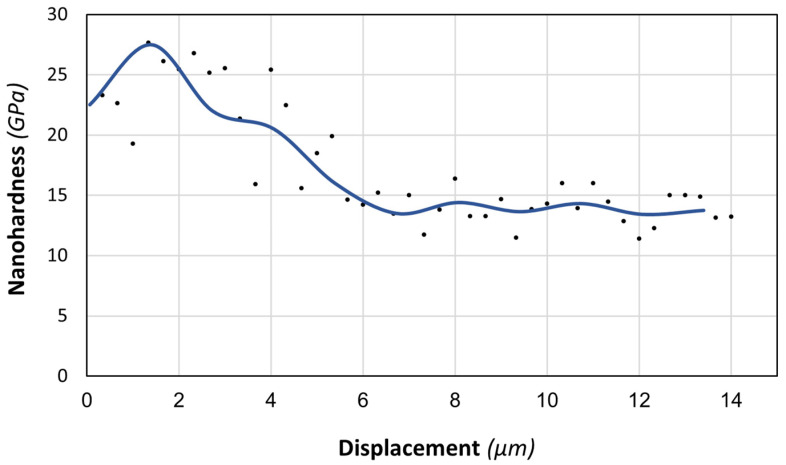
Nanohardness distribution in the cross-section of the 282–AB+N specimen with the layer containing CrN + Cr_2_N phases.

Nanohardness distribution analysis along the cross-section in the near-surface zone showed a strengthening of the material after ion nitriding to a depth of about 7 μm (Figure 8), which corresponds to the layer thickness determined using microscopic observations (Figure 5). The hardness of the core of the 282–AB+N specimen reaches 13.5 ± 1.9 GPa. As it approaches the surface, it smoothly increases to a maximum value of about 27 GPa at a depth of about 1.4 μm, followed by a slight decrease associated with the morphology of the surface zone of the nitrided layer.

### 3.4. Corrosion Resistance

**Figure 9:**
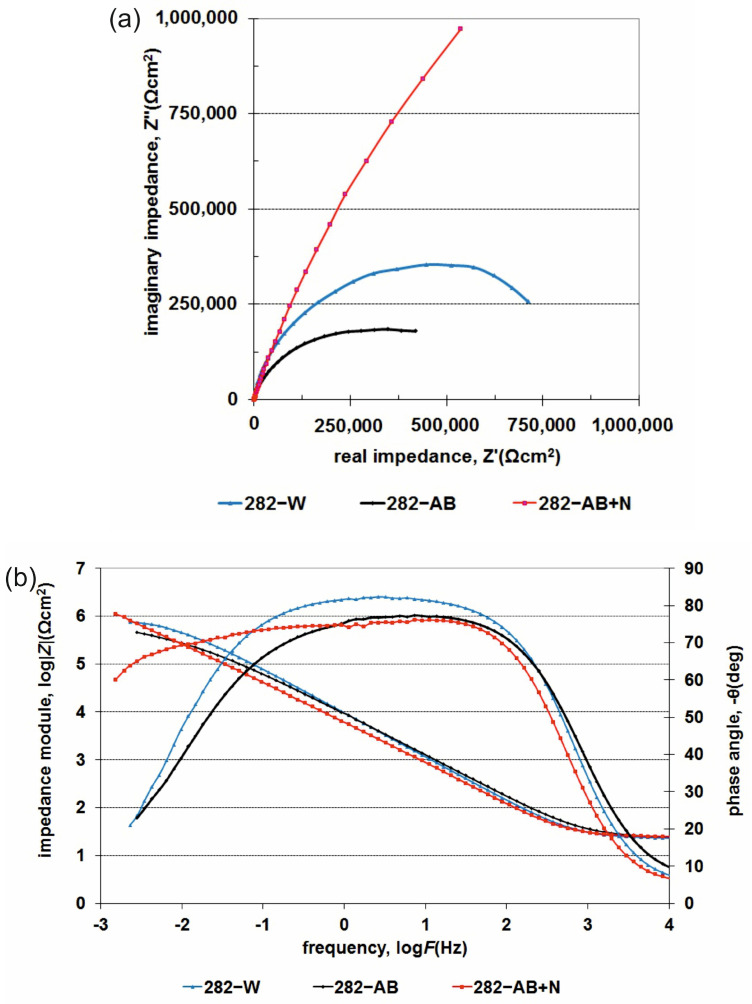
Impedance spectra: (**a**) Nyquist and (**b**) Bode diagrams for 282–W, 282–AB and 282–AB+N specimens.

**Figure 10:**
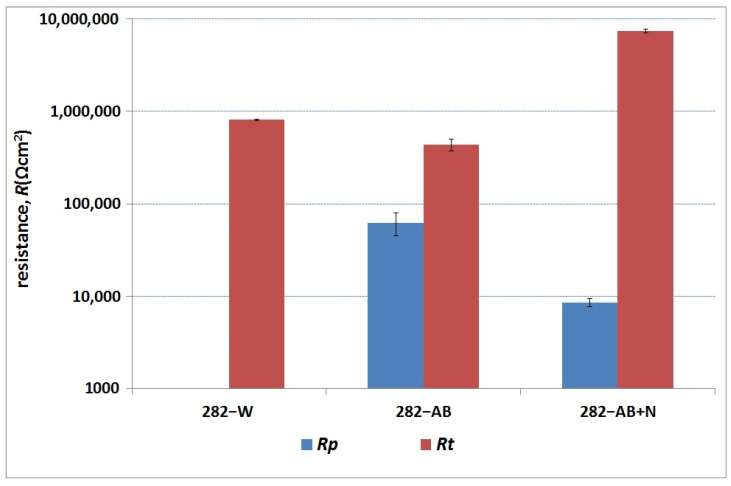
Resistance value of dielectric (R_p_) and double layer (R_t_).

For the 282–W specimen, an electrical replacement circuit (EC) with a one-time constant R(RQ) (where R is resistance and Q is capacity; see Table 3 below) was used, while the 282–AB and 282–AB+N employed a replacement circuit with two-time constants R(RQ)(RQ). Two-time constants in the starting material are conditional on a heterogeneous substrate structure, typical for materials after laser processing, in which the paths of penetration of the substrate with a laser beam and familiar ‘tears’ are observed. The production of a layer containing chromium nitrides on a substrate produced the DMLS technique significantly modified the parameters and electrochemical nature of the substrate due to numerous nitride precipitates on the surface and some surface layer discontinuities as observed in the microscopy study. They were analyzed using a replacement system R(Q[R(RQ)]), which is commonly applied for materials susceptible to local corrosion. The resulting spectra for specimens of 282 alloy in three states are presented in the form of Bode and Nyquist graphs in Figure 9. The analysis of the spectra and data presented in Table 3 and Figure 10 confirmed the positive effect of the nitrided layer on the corrosion resistance of 282 alloy.

The increase of the charge transfer resistance through the double layer (resistance R_t_) from 4.4 × 10^5^ Ωcm^2^ (282–AB) to 7.4 × 10^6^ Ωcm^2^ for 282–AB+N led to a significant enhancement of the corrosion resistance. The data analysis in Table 3 and Table 4 indicates a development of a roughened and porous CrN + Cr_2_N layer that increased the active surface several times. This is indicated by the values of the parameter ‘n’ of the double layer (n = 0.66) and the parameter S_a_ (S_a_ = 118 nm) of the surface roughness (Table 5). Both of these values indicate a significant share of diffusion factors determining the material’s corrosion resistance.

However, the presence of a dielectric layer with a reduced resistance (8.6 × 10^3^ Ωcm^2^), and a slight decrease in the value of the parameter n (0.89) indicate an increasing surface roughness as well as local differences in the rates of electrode reactions occurring on the surface or micro areas with differences in the chemical composition. The substrate and nitride layer’s roughness values are presented in Table 5. All of these factors may imply local discontinuities facilitating the degradation and initiation of the pitting corrosion observed in potentiodynamic studies.

**Figure 11:**
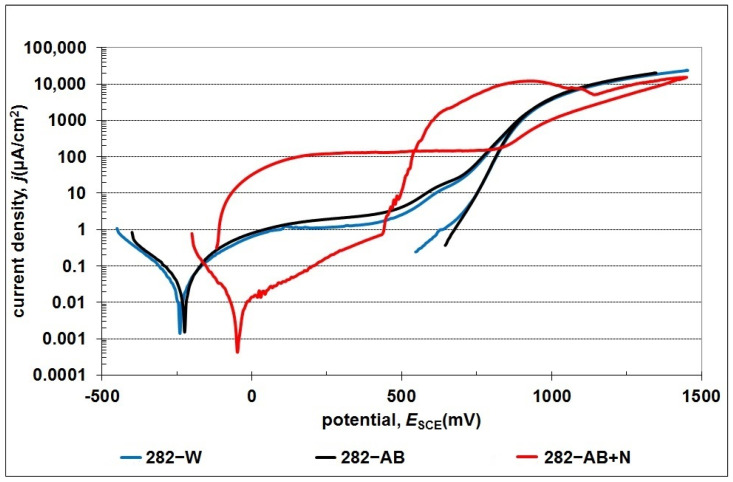
Potentiodynamic curves obtained for the 282–W, 282–AB, and 282–AB+N specimens.

**Figure 12:**
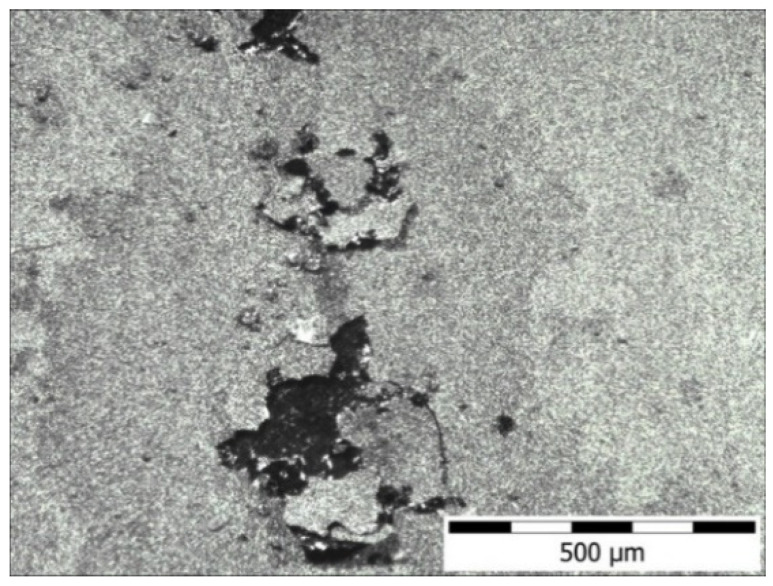
Surface topography of 282–AB+N specimen after potentiodynamic tests.

The potentiodynamic curves in Figure 11 and the results summarized in Table 4 confirmed the positive effect of the nitride layer on the corrosion resistance of the 282–AB+N specimen. This is reflected in the decrease of the density values of corrosion currents from 4.09 × 10^−2^ μA/cm^2^ (282–AB) to 8.89 × 10^−3^ μA/cm^2^ and an increase in the value of corrosion potentials. The change in the electrochemical parameters for the 282–AB+N specimen results can be explained by a partially ceramic character of the nitride layer that lowers conductivity and increases corrosion potential. However, when the pitting potential is reached, the surface does not have the repassivation capacity shown by the non-nitrided alloys, since the Cr is combined with the N, and, therefore, the pits formed on the surface can grow. In the case of the 282–AB+N specimen, the observed constant increase in the current density that accompanies the polarization intensity increase (up to approx. 400 mV) is typical for nitride layers produced on metallic substrates. This result is associated with the presence of nitrogen in the top layer. The presence of nitrogen and its susceptibility to oxidation facilitates the oxidation of the superalloy during an anodic polarization. Taguchi and Kurihara’s research has additionally shown that chromium loses its self-passivation capacity during nitriding [56]. An increase in the intensity of polarization results in the initiation of pitting corrosion (E = 430 mV, Enp—breakdown potential initiating pitting corrosion) and flaking of the nitrided layer (Figure 12). A further increase in the intentionality of polarization allows the start of another electrochemical process associated with releasing oxygen from the solution and the decomposition of water. The reverse polarization process showed the existence of a large hysteresis loop and the absence of repassivation potential. This result indicates that the damaged nitrided layer does not undergo spontaneous repassivation and is completely degraded by its exfoliation. This result shows that the possible application of nitrided layers can be proposed for conditions that do not damage mechanical nitrided layers or for chloride-free environments.

In the case of 282–W and 282–AB specimens, regardless of the structure, the surfaces remain passive in a wide range of tested potentials. The data analysis in Table 3 indicates a slight decrease (by approximately 5%) in the corrosion resistance of the 282–AB compared to the 282–W. In both cases, the durability of passive layers and the current densities in the passive area have been compared. The observed increase in the current density at approx. 500 mV is caused, according to Pourbaix charts, by a change in the oxidation stage of nickel (at approx. 500 mV) and a change in the oxidation stage of chromium (at approx. 650 mV), i.e., the main alloying components of the 282–W. In the case of nickel, the oxidation from Ni(OH)_2_ to Ni_3_O_4_ occurs in the potential of about 500 mV [57], while a further increase in current density is caused by the corrosion of passive chromium oxide Cr_2_O_3_ to the soluble form of CrO_4_^2−^. The formation of a layer of corrosion products and the achievement of a diffusion limit current by the system stabilizes the intensity of the electrochemical processes.

Based on the absence of a typical hysteresis loop on the return polarization curves, the observed increase in the current density above 500 mV can be associated with the trans-passive region, in which the intensity of processes is related to the oxidation of the substrate and a release of oxygen from the solution.

### 3.5. Tensile Testing at 750 °C

**Figure 13:**
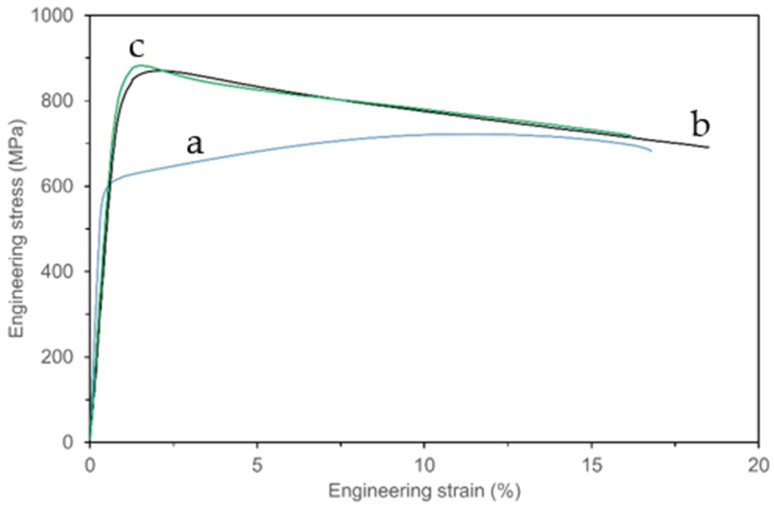
Development of stress–strain curves for the specimens: 282–W—(a), 282–AB—(b), and 282–AB+N—(c).

Based on the testing of the mechanical behavior at 750 °C carried out for 282–W, 282–AB, and 282–AB+N (Figure 13), it can be concluded that 282-AB and 282–AB+N specimens showed comparable yield strength (σ_y_) as well as ultimate tensile stress (UTS) with very low influence of the nitride layer. The values of σ_y_ and UTS for 282–W were lower, as listed in Table 6. It is remarkable that the specimens built with DMLS showed a significant decrease in strength during tensile testing at 750 °C, with very small plastic deformations after reaching the yield strength (curve b and c in Figure 13).

Differences in the mechanical behavior and strength of the tested specimens are caused by their microstructure features. The 282–W specimens were thermo-mechanically treated, reinforced by γ′ particles and carbides, while the 282–AB and 282–AB+N specimens show anisotropic character due to the high cooling rates at the DMLS building. Therefore, it can be considered that they are in a supersaturated solid solution state and contain just a little or no content of the γ′ strengthening phase and carbides. However, some defects, e.g., micropores and dislocations in the built structure, were detected. Similar values for the nitrided specimen compared to the 282–AB suggest that the heat conditions of the nitriding process did not significantly affect the phase precipitations in the substrate. The layer containing CrN + Cr_2_N phases is too thin compared to the cross-section of the tested specimens and does not have a significant effect on the tensile strength at 750 °C.

## 4. Conclusions

Based on the research carried out for 282 alloy specimens in the conditions of wrought (282–W), as-built using DMLS technique (282–AB), or as-built and treated with the nitriding process (282–AB+N), the following conclusions can be drawn:

1. As-built specimens were characterized by a high density of the dislocations arranged to the cells depending on the anisotropic structure due to the scanning strategy.
2. Ion nitriding of as-built specimen at 570 °C leads to the formation of an approximatively 7 μm thick continuous layer with good adherence to the substrate.
3. A very fine surface film of the nitrided layer contained nanocrystals of CrN/Cr_2_N phases.
4. Residual tensile stresses were found to be higher by 88 MPa in the specimens as-built with DMLS compared to the as-built and nitrided specimens.
5. Specimens protected by nitrided layer demonstrated high corrosion resistance.
6. Nitrided layer showed a negligible effect on the yield strength, ultimate tensile stress, and elongation.
7. Tensile behavior of the specimens built with DMLS differs from the purchased 282 alloy due to the microstructure characteristics.

## Figures and Tables

**Table 1:** Chemical composition of Amperprint^®^0233 Haynes^®^ 282^®^ powder (% wt.) [41].

Ni	Cr	Co	Mo	Ti	Al	Fe	Mn	Si	C	B
Bal	19.5	10.0	8.5	2.1	1.5	1.5 *	0.3 *	0.15 *	0.05	0.005

* max. content of element.

**Table 2:** Chemical composition of purchased wrought Haynes^®^ 282^®^ alloy (% wt.) [42].

Ni	Cr	Co	Mo	Ti	Al	Fe	Mn	Si	C	B
Bal.	20	10.0	8.5	2.1	1.5	1.5 *	0.3 *	0.15 *	0.06	0.005

* max. content of element.

**Table 3:** Characteristic electrochemical values of tested specimens (impedance tests).

	Substitute Arrangement		Dielectric Layer	Err %	Double Layer	Err %
282–W	R(RQ)	R(Ωcm^2^)			8.07 × 10^5^	1.3
		Q _CPE_(Fcm^−2^ × s^n−1^)			1.99 × 10^−5^	0.5
		n			0.91	0.1
282–AB	R(RQ)(RQ)	R(Ωcm^2^)	6.26 × 10^4^	27.5	4.36 × 10^5^	15.7
		Q _CPE_(Fcm^−2^ × s^n−1^)	4.99 × 10^−5^	20.3	3.87 × 10^−5^	35.9
		n	0.86	1.2	0.88	6.4
282–AB+N	R(Q[R(RQ)])	R(Ωcm^2^)	8.61 × 10^3^	10.8	7.43 × 10^6^	4.6
		Q _CPE_(Fcm^−2^ × s^n−1^)	2.67 × 10^−5^	1.2	1.03 × 10^−5^	2.8
		n	0.89	0.2	0.66	0.5

R—resistance, Q _CPE_—capacity of constant phase element, n—coefficient of imperfections of constant phase element (CPE); an empirical constant ranging from 0 to 1. It is worth noting that when n = 1, the CPE behaves as a pure capacitor, while when n = 0, the CPE behaves as a pure resistor [55].

**Table 4:** Characteristic electrochemical values of the tested specimens.

	282–W	SD (±)	282–AB	SD(±)	282–AB+N	SD (±)
R_p_ (kΩcm^2^)	809	24	637	11	2763	96
I_corr_ (µA/cm^2^)	3.88 × 10^−2^	0.002	4.09 × 10^−2^	0.002	0.89 × 10^−2^	0.0007
E_corr_ (mV)	−240	10	−225	25	−45	15

where: R_p_—polarization resistance; I_corr_—corrosion current density; E_corr_—corrosion potential.

**Table 5:** Surface roughness parameters of the tested specimens [in nm].

	S_a_	SD	R_a_	SD	R_z_	SD
282–W	12.78 ± 0.15		7.35 ± 0.62		76.14 ± 13.74	
282–AB	16.45 ± 0.24		6.39 ± 0.40		71.82 ± 10.63	
282–AB+N	118.93 ± 1.37		84.25 ± 5.26		1186.65 ± 263.02	

Where S_a_—average arithmetic deviation of the roughness surface from the median line; R_a_—average arithmetic deviation of the roughness profile from the median line along the elementary length (2D tests); R_z_—distance from the highest point of the roughness profile to its lowest point measured along the elementary length (2D tests); SD—standard deviation.

**Table 6:** Average mechanical properties of the tested specimens.

Specimens	σ_y_ (MPa)	UTS (MPa)	A (%)
282–W	604 ± 4	715 ± 4	16 ± 1.5
282–AB	810 ± 3	870 ± 4	17 ± 6.4
282–AB+N	830 ± 5	882 ± 6	15 ± 8.5

## Data Availability

Data are available from the first author and can be shared with anyone upon reasonable request.
